# Distinct chemical structures inhibit the CEMIP hyaluronidase and promote oligodendrocyte progenitor cell maturation

**DOI:** 10.1016/j.jbc.2024.107916

**Published:** 2024-10-24

**Authors:** Alec Peters, Fatima Banine, Kanon Yasuhara, Angela Hoffman, Basappa, Prashant K. Metri, Lily Gunning, Ava Huffman, Jake VanCampen, Clinton C. Shock, Stephen A. Back, Larry S. Sherman

**Affiliations:** 1 Division of Neuroscience, Oregon National Primate Research Center, Beaverton, Oregon, USA; 2 Department of Cell, Developmental and Cancer Biology, Oregon Health & Science University, Portland, Oregon, USA; 3 Department of Chemistry, University of Portland, Portland, Oregon, USA; 4 Department of Studies in Organic Chemistry, University of Mysore, Manasagangotri, India; 5 College of Agricultural Sciences, Oregon State University, Corvallis, Oregon, USA; 6 Departments of Pediatrics and Neurology, Oregon Health & Science University, Portland, Oregon, USA

**Keywords:** hyaluronan, hyaluronidase, CEMIP, flavonoids, sulfuretin, oligodendrocytes

## Abstract

Growing evidence supports pathogenic roles for chronically elevated hyaluronidase activity in numerous conditions. Elevated expression of one such hyaluronidase, the Cell Migration Inducing and hyaluronan binding Protein (CEMIP), has been implicated in the pathogenesis and progression of several cancers as well as demyelinating diseases in the central nervous system (CNS). Developing effective and selective CEMIP inhibitors could therefore have efficacy in treating a variety of conditions where CEMIP is chronically elevated. Using two distinct screens for novel hyaluronidase inhibitors, we identified two synthetic thiocarbamates and one plant-derived flavonoid, sulfuretin, that effectively blocked CEMIP activity in live cells, including a tumorigenic cell line and primary cultures of oligodendrocyte progenitor cells (OPCs). None of these agents influenced cell proliferation, but they had differential dose-dependent and cell type-specific effects on cell survival. Furthermore, we found that each of these agents could promote oligodendrocyte maturation by OPCs in the presence of high molecular weight (>2 Mda) hyaluronan, the accumulation of which is linked to the inhibition of OPC maturation and remyelination failure in demyelinating diseases. These findings indicate that CEMIP can be inhibited through distinct chemical interactions and that CEMIP inhibitors have potential efficacy for treating demyelinating diseases or other conditions where CEMIP is elevated.

Dynamic changes in the composition of the extracellular matrix (ECM) are implicated in the regulation of a wide variety of cellular activities including cell division, migration, differentiation, and survival. For example, the ECM can influence the behaviors of cells during embryonic development but also the proliferation and metastasis of cancer cells (1). In the central nervous system (CNS), alterations in the ECM contribute to neuroplasticity but can also limit repair processes in neurodegenerative diseases (1, 2, 3, 4).

Glycosaminoglycans and proteoglycans are major components of the ECM that can influence each of these cellular processes. In particular, increased synthesis of the extracellular glycosaminoglycan hyaluronan (also called hyaluronic acid; HA), accompanied by HA catabolism through hyaluronidase activity, is associated with cancer progression and poor prognosis, as well as delayed recovery in CNS insults, including multiple sclerosis (MS) and ischemic injury (5, 6). These pathological effects are linked to the accumulation of HA fragments of various sizes that have distinct biological activities (6). Inhibiting hyaluronidase activity using a small molecule-based therapy may therefore be a tractable method to improve patient outcomes in cancer, CNS diseases, and other conditions.

Mammals possess multiple hyaluronidases that have distinct activities, localizations, and mechanisms of HA catabolism (7). These include six homologs of one of the first identified hyaluronidases, bee venom hyaluronidase, called HYAL1, HYAL2, HYAL3, HYAL4, HYAL6P/HYALP1, and PH20, and two unrelated G8-domain-containing proteins called Cell Migration Inducing and hyaluronan binding Protein (CEMIP, also called KIAA1199), and transmembrane protein-2 (TMEM2, also called CEMIP2) that digest HA through different mechanisms from the other hyaluronidases. Elevated expression of CEMIP has been associated with increased metastasis and poor prognosis in multiple cancer types, including colorectal (8, 9), thyroid (10), gastric (11), and hepatic cancers (12, 13). Furthermore, CEMIP expression in tumor exosomes increases metastases to the brain in mice, and increased CEMIP expression in human tumors is associated with increased numbers of brain metastases in patients (14). CEMIP has also been implicated in the pathogenesis of multiple sclerosis (MS), a disease characterized by inflammatory demyelination in the CNS, leading to sensory, motor, and cognitive dysfunction (15, 16). Chronically demyelinated MS lesions exhibit an accumulation of oligodendrocyte progenitor cells (OPCs) that fail to differentiate into myelinating oligodendrocytes. This is in part due to elevated hyaluronidase activity, producing bioactive HA fragments that directly inhibit OPC differentiation (17, 18, 19). CEMIP expression is reported to be elevated in demyelinating MS lesions (20), and an inhibitor that blocks the activities of some hyaluronidases, including CEMIP, increased functional recovery in a rodent model of MS (21). Targeting CEMIP hyaluronidase activity with therapeutic small molecule inhibitors may therefore be an efficacious method to promote repair following CNS insults, reduce cancer cell metastasis to the brain, and reverse other pathological conditions linked to elevated HA catabolism.

Numerous hyaluronidase inhibitors have been characterized with varying degrees of efficacy in blocking HA digestion (22). These include synthetic and plant-derived compounds, proteins, polysaccharides, fatty acids, and glycosaminoglycans. Among plant-derived inhibitors, flavonoids, their biosynthetic precursors chalcones, and the closely related compound class of aurones have been found to have anti-cancer, anti-inflammatory, and hyaluronidase inhibitory activities (23, 24, 25, 26, 27, 28). Recently, we described a structural derivative of the flavone apigenin that selectively inhibited CEMIP activity over other hyaluronidases (21). Named S3, the flavone was also found to accelerate functional remyelination in a mouse model of demyelinating disease (21).

Although S3 inhibited hyaluronidase activity, it only poorly crosses the blood-brain barrier, is relatively insoluble, and requires relatively high doses to block CEMIP activity. S3, therefore, is unlikely to be useful as an intravenous or oral drug to treat elevated CEMIP activity that contributes to disease pathogenesis. Here, we took a two-pronged approach to identify novel CEMIP inhibitors to better understand the chemistry of hyaluronidase inhibitors and to identify potential lead compounds for therapeutic use. We screened a library of novel, synthetic thiocarbamates and also screened a large number of plant extracts for novel agents that block CEMIP activity. We compared the agents we identified to S3 with regards to their anti-hyaluronidase activity in both a tumorigenic cell line and in primary cultures of OPCs, then examined the best of these compounds for their ability to influence cell proliferation, survival, and differentiation.

## Results

### Identification of novel hyaluronidase inhibitors

**Figure 1:**
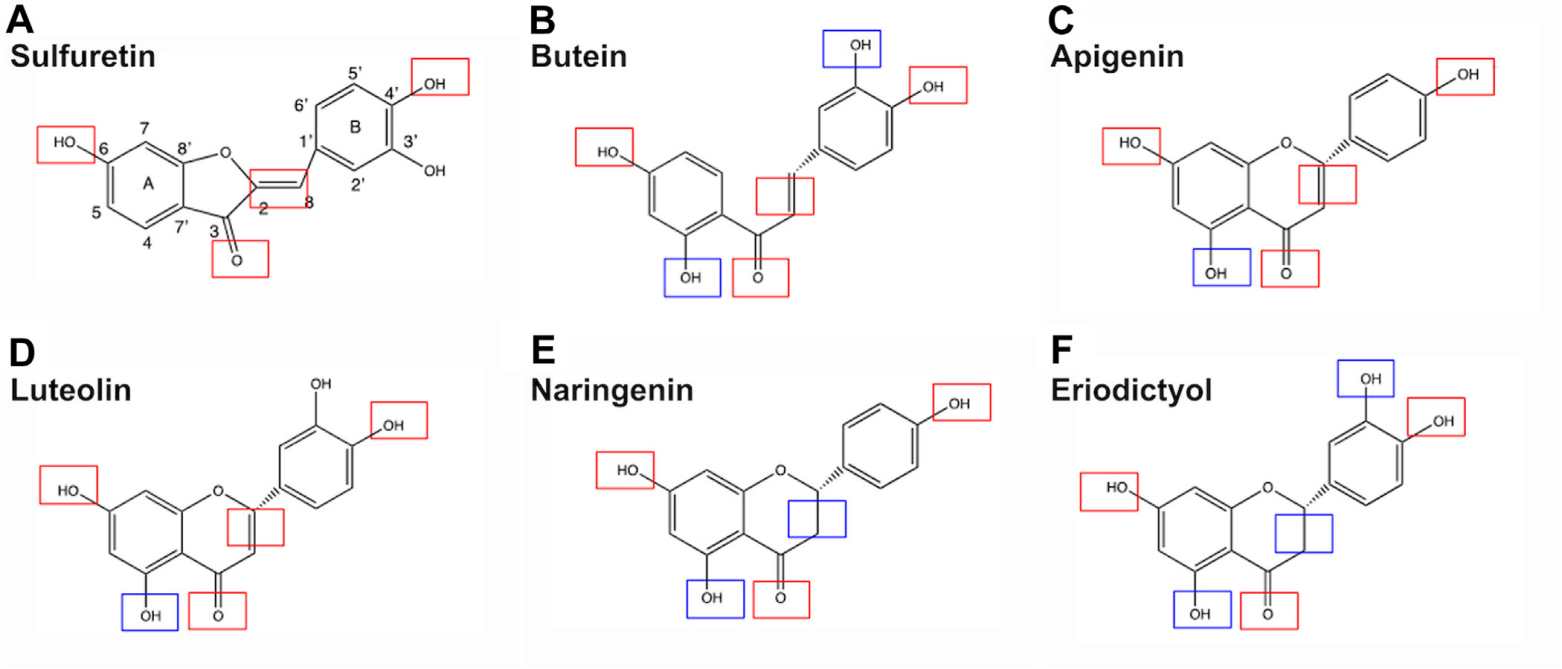
**Structures of flavonoids extracted from dahlia and coreopsis flowers.** (*A*) *Red boxes* denote similarities between compound structures and sulfuretin, while *blue boxes* denote differences. The other structures are (*B*) butein, a chalcone; (*C*) apigenin, a flavone that has previously been used as a hyaluronidase inhibitor; (*D*) luteolin, another flavone; (*E*) naringenin, a compound in the flavonoid group of polyphenols; (*F*) eriodictyol, another flavone.

To understand the range of chemical structures that can block CEMIP activity, we first screened for naturally occurring agents that could block the activity of a similar hyaluronidase, bovine testicular hyaluronidase, the activity of which is mostly linked to the PH20 hyaluronidase in sperm (29, 30, 31). Bovine testicular hyaluronidase (50 U/ml) was mixed with high molecular weight (HMW) HA (>1 MDa) as a substrate along with fractionated extracts from a variety of plants. The extracts were tested for their ability to block HA digestion as assessed by gel electrophoresis and labeling with Stains-All as previously described (21). Strong inhibitors were defined as agents that blocked >90% of hyaluronidase activity. We identified several fractions from different plants that were able to block hyaluronidase activity (Table 1). Using silica gel thin-layer chromatography, we confirmed that the most active agents in each of the fractions were flavonoids. The most active fraction was isolated from compound F and was identified as the aurone sulfuretin (Fig. 1*A* and Table 2; structure confirmed by NMR, see Table 3) which had not previously been isolated from coreopsis (tickseed) flowers. Previous studies suggested that sulfuretin has anti-inflammatory, neuroprotective, and anti-cancer activities (32, 33, 34, 35, 36), but none had supported a role for sulfuretin as a hyaluronidase inhibitor. Fractions from dahlia plants (compounds J, K, M; Table 1) also had higher activity compared to other fractions from other plants and included the previously identified weaker hyaluronidase inhibitors luteolin (which was also confirmed by NMR; Table 3), apigenin, butein, and eriodictyol (23, 37). Consistent with one previous report (38), we found that naringenin also had weak hyaluronidase inhibitory activity, although another report indicated that naringenin did not inhibit hyaluronidases found in venoms (39).

**Table 1:** Extracts and plant sources

Extract	Source
A	Primula
B	Apple
C	Rose
D	Geranium
E	Glycyrrhiza
F	Coreopsis
G	Marigold
H	Alfalfa
I	Columbine
J	Dahlia
K	Dahlia
L	Columbine
M	Dahlia

**Table 2:** Flavonoids isolated from dahlia and coreopsis that strongly inhibit hyaluronidase activity

Compound name	Flavonoid class	Molecular formula	Molecular weight	CAS number
Sulfuretin	Aurone	C_15_H_10_O_5_	270.24	120-05-8
Luteolin	Flavone	C_15_H_10_O_6_	286.24	471-70-3
Butein	Chalcone	C_15_H_12_O_5_	270.25	487-52-5
Apigenin	Flavone	C_15_H_10_O_5_	270.24	520-36-5
Naringenin	Flavanone	C_15_H_12_O_5_	272.26	480-41-1
Eriodictyol	Flavanone	C_15_H_12_O_6_	288.26	552-58-9

**Table 3:** ^1^H NMR and ^13^C NMR characterization of sulfuretin and luteolin

Name	Description	MW (Da)	^1^H-NMR	^13^C-NMR
Sulfuretin	Orange powder	270	(DMSO-d6) δ: 6.64 (1H, s, H-2), 6.70 (1H, dd, J = 1.85, 8.25 Hz, H-6), 6.74 (1H, d, J = 1.85 Hz, H-8), 6.83 (1H, d, J = 8.25 Hz, H-5), 7.24 (1H, dd, J = 2.3, 8.2 Hz, H-6), 7.44 (1H, d, J = 1.85 Hz, H-2), 7.60 (1H, d, J = 8.7 Hz, H-5)	(DMSO-d6) δ: 112.3 (C-2), 146.2 (C-3),148.6 (C-4), 126.3 (C-5), 125.1 (C-6), 168.0 (C-7), 98.9 (C-8),166.7 (C-9), 113.8 (C-10), 123.9 (C-1), 118.5 (C-2), 146.1 (C-3), 148.6 (C-4), 116.6 (C-5), 125.1 (C-6)
Luteolin	Light yellow powder	287	(CD_3_OD) δ 6.19 (1H, d, J = 2.0 Hz, H-6), 6.42 (1H, d, J = 2.0 Hz, H-8), 6.52 (1H, s, H-3), 6.88 (1H, d, J = 8.5 Hz, H-5′), 7.36 (2H, m, H-2′, H-6′)	(CD_3_OD) δ 95.0 (C-8), 100.1 (C-6), 103.9 (C-3), 105.3 (C-10), 114.1 (C-2′), 116.8 (C-5′), 120.3 (C-6′), 123.7 (C-1′), 147.0 (C-3′), 151.0 (C-4′), 159.4 (C-9), 163.2 (C-5), 166.0 (C-2), 166.4 (C-7), 183.9 (C-4)

Analysis of the chemical structures of these agents reveals multiple similarities and differences between the structures (Fig. 1, *A*–*F*). These data indicate that the hydroxyl groups on carbons 6 and 4′ and the carbonyl group on carbon 3 appear to be essential for inhibiting hyaluronidase activity. A double bond between carbons 2 and 3, as well as hydroxyl groups on carbons 4 and 5′ may also be involved in biological activity, but are non-essential.

**Figure 2:**
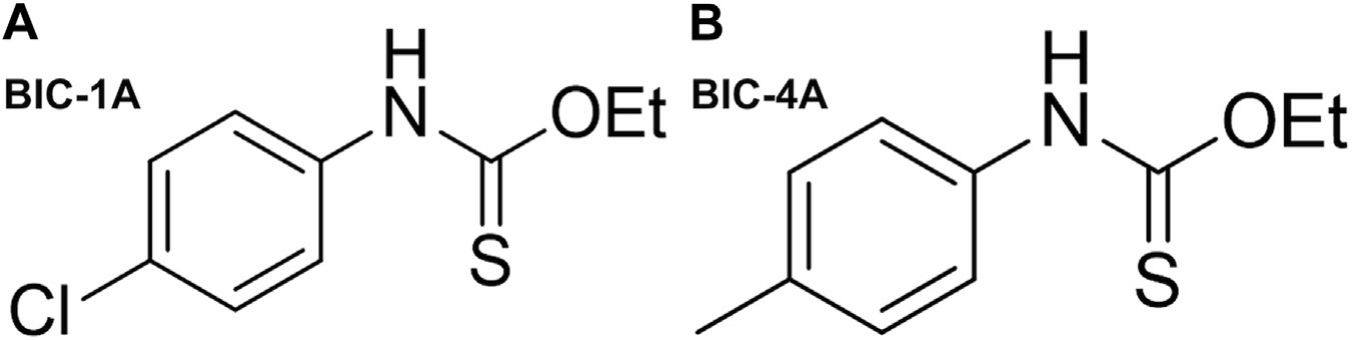
**Chemical structures of thesynthetically-derivedthiocarbamates.** (*A*) BIC-1A and (*B*) BIC-4A.

As a complementary strategy to our screening of plant extracts, we screened a compound library of thiocarbamates, a family of organosulfur compounds, for additional molecules with hyaluronidase inhibitor activity. We chose to focus on thiocarbamates because previous studies had demonstrated that these chemicals can be potent α-glucosidase inhibitors. For example, coumarin-based dithiocarbamates have significant α-glucosidase inhibitory activity (40). In addition, ethanol extracts of thiocarbamate-based glycosides, including niazidin and its derivatives, have been found to have activity (41). We therefore synthesized thiocarbamate libraries and screened them for hyaluronidase inhibitory activity as previously described (21). We identified two molecules from this screen, BIC-1A and BIC-4A, that had strong inhibitory activity that was equivalent or superior to sulfuretin (Fig. 2, *A* and *B*). Based on these results, we further characterized sulfuretin, BIC-1A, and BIC-4A for their hyaluronidase inhibitory activity in live cells.

### Both synthetic and natural hyaluronidase inhibitors block CEMIP activity in live cells

**Figure 3:**
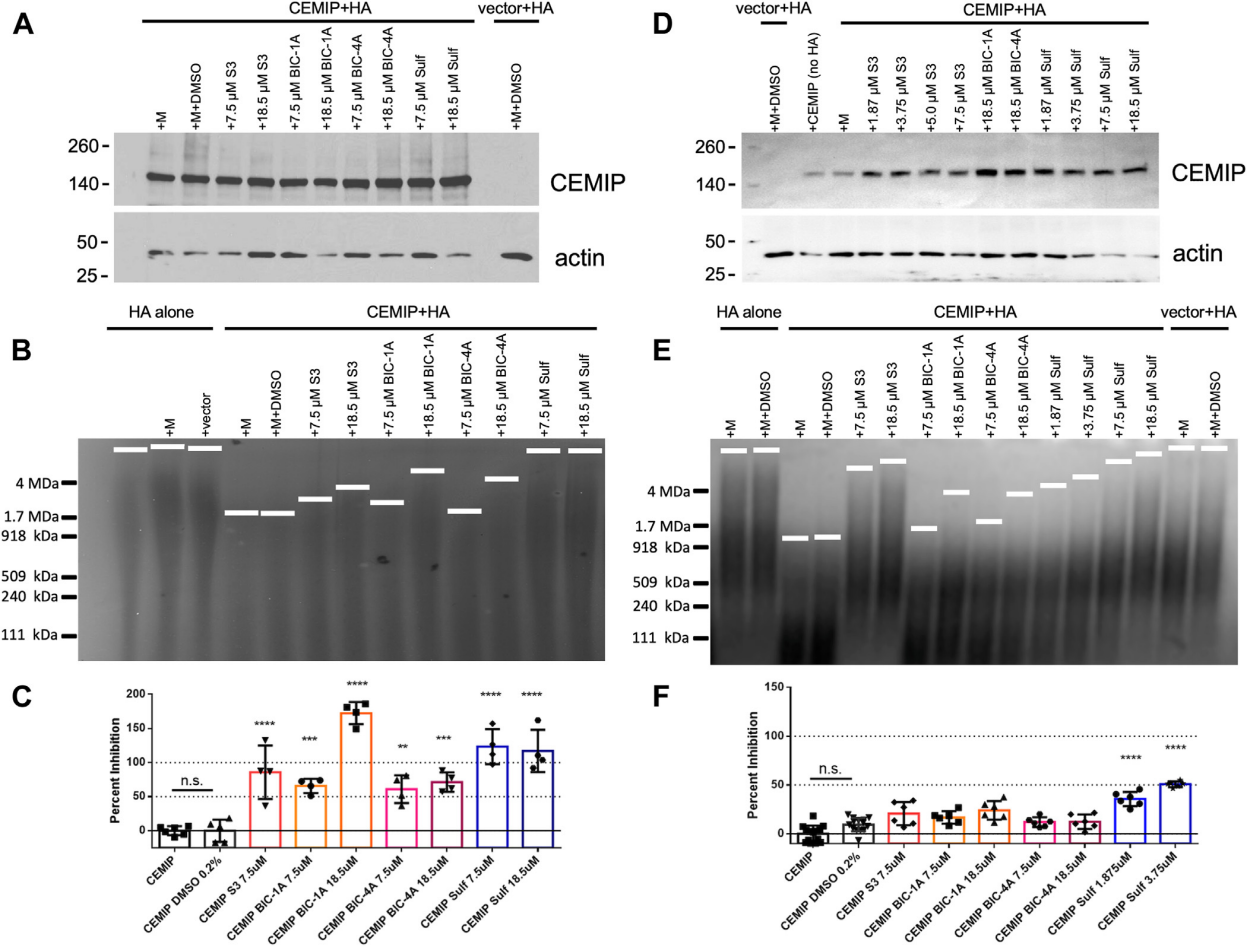
**Novel synthetic flavonoids and sulfuretin inhibit CEMIP activity.***A*, Western blot showing CEMIP expression in 293T cells transfected with a CEMIP-expression vector. Actin was used as a loading control. *B*, representative HA agarose gel shows that 293T cells overexpressing CEMIP degrade HMW HA and that S3, BIC-1A, BIC-4A, and sulfuretin block this hyaluronidase activity in a dose-dependent manner. Note that the slight differences in the sizes of HMW HA in media alone compared to the vector control are within the range of normal HMW HA size variability. *C*, quantification of inhibitory activity of each compound in comparison to S3 in 293T cells. *D*, Western blot showing CEMIP expression in primary cultures of OPCs transfected with a CEMIP-expression vector. *E*, representative HA agarose gel showing that OPCs overexpressing CEMIP degrade HMW HA and that the hyaluronidase inhibitors block this activity. *F*, quantification of inhibitory activity of each compound in comparison to S3 in OPCs. ∗∗*p* < 0.01, ∗∗∗*p* < 0.001, ∗∗∗∗*p* < 0.0001, and n.s. – not significant.

We tested the CEMIP hyaluronidase inhibitory activity of sulfuretin, BIC-1A, and BIC-4A at different concentrations in live tumorigenic 293T cells and primary cultures of OPCs transfected with a CEMIP expression vector compared to untransfected cells and cells transfected with an empty vector. We also compared these agents with the activity of the S3 hyaluronidase inhibitor as previously described (21). We found that sulfuretin, BIC-1A, and BIC-4A demonstrated inhibitory activity that is superior to S3 (Fig. 3). In the 293T cell line, we found that BIC-1A and sulfuretin were both potent CEMIP hyaluronidase inhibitors (65%, *p* < 0.001; 123%, *p* < 0.0001 respectively at 7.5 μM; Fig. 3, *A*–*C*). Surprisingly, in primary oligodendrocyte progenitor cells (OPCs), sulfuretin potently inhibited CEMIP hyaluronidase activity at even lower concentrations than in 293T cells (95%, *p* < 0.0001 at 7.5 μM), while BIC-1A, and BIC-4A were less effective (24%, *p* < 0.01; 12%, *p* = n.s. respectively at 18.5 μM; Fig. 3, *D*–*F*).

**Figure 4:**
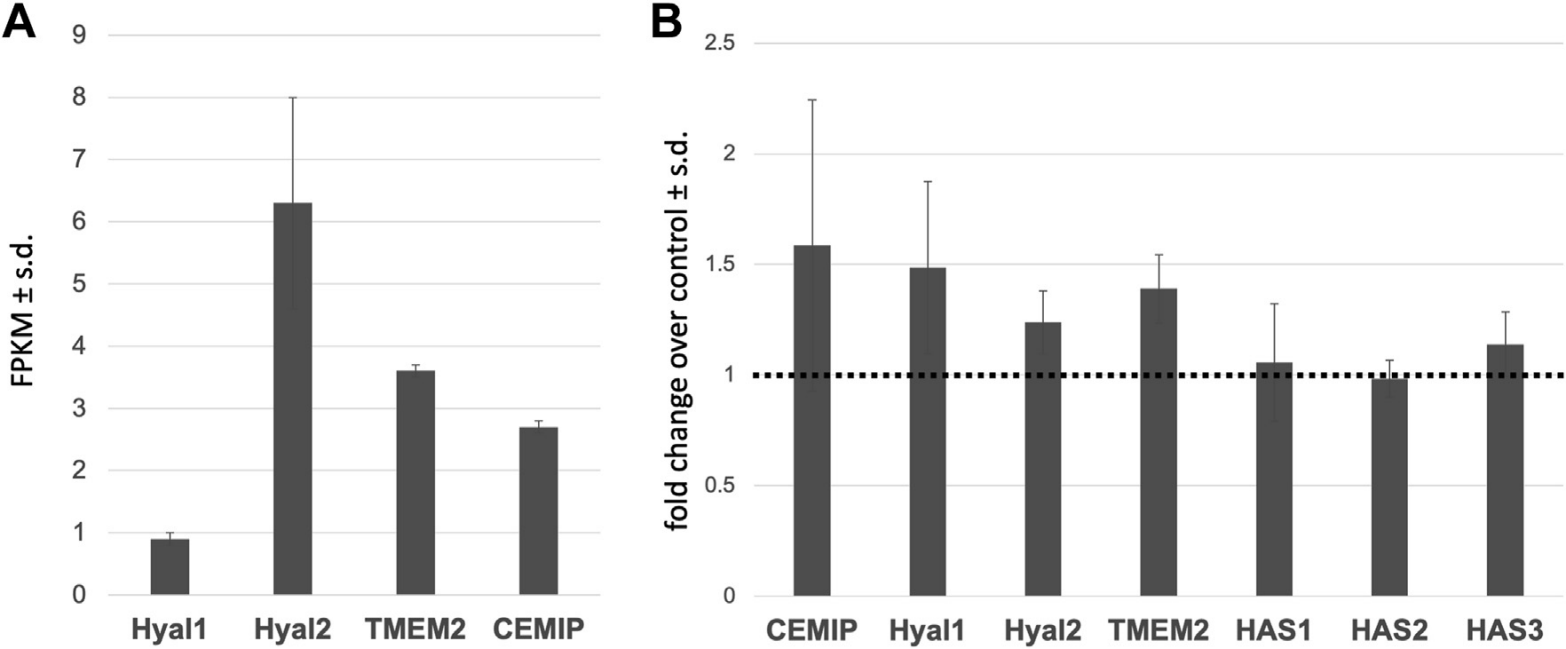
**Sulfuretin does not influence hyaluronidase expression in OPCs.***A*, relative expression (fragments per kilobase of transcript per million fragments mapped; FPKM) of hyaluronidase transcripts in mouse OPCs. Data were collected from the Brain RNA-Seq database (https://brainrnaseq.org). *B*, qPCR analysis of hyaluronidase and HA synthase transcripts in OPCs treated with 3.75 μM sulfuretin for 24 h compared to vehicle (DMSO)-treated controls (represented by the *dashed line*). There was no significant difference in expression of any of the genes analyzed.

Although, as described earlier, CEMIP is elevated compared to other hyaluronidases in some cancer cells and in demyelinating lesions, it is possible that the inhibitors tested here can act by influencing the expression of multiple hyaluronidases or HA synthases. Given the potent effects of sulfuretin on OPCs, previous findings showing that OPCs have hyaluronidase activity (42, 43, 44), and the finding that blocking this activity can promote OPC maturation and remyelination (21), we assessed which hyaluronidases are expressed by OPCs and whether sulfuretin influences the expression of these hyaluronidases or HA synthases. We used the Brain RNAseq database (https://brainrnaseq.org) to assess the relative expression levels of *Hyal1*, *Hyal2*, *TMEM2* and *CEMIP* in OPCs. We find that *Hyal1* is weakly expressed by OPCs while *Hyal2*, *TMEM2*, and *CEMIP* all have higher levels of expression (Fig. 4*A*). Following treatment with sulfuretin, there was a non-significant trend towards increased *CEMIP* and *Hyal1* expression, with little to no change in the expression of other hyaluronidases or HA synthases (Fig. 4*B*).

### CEMIP inhibitors differentially influence cell survival

**Figure 5:**
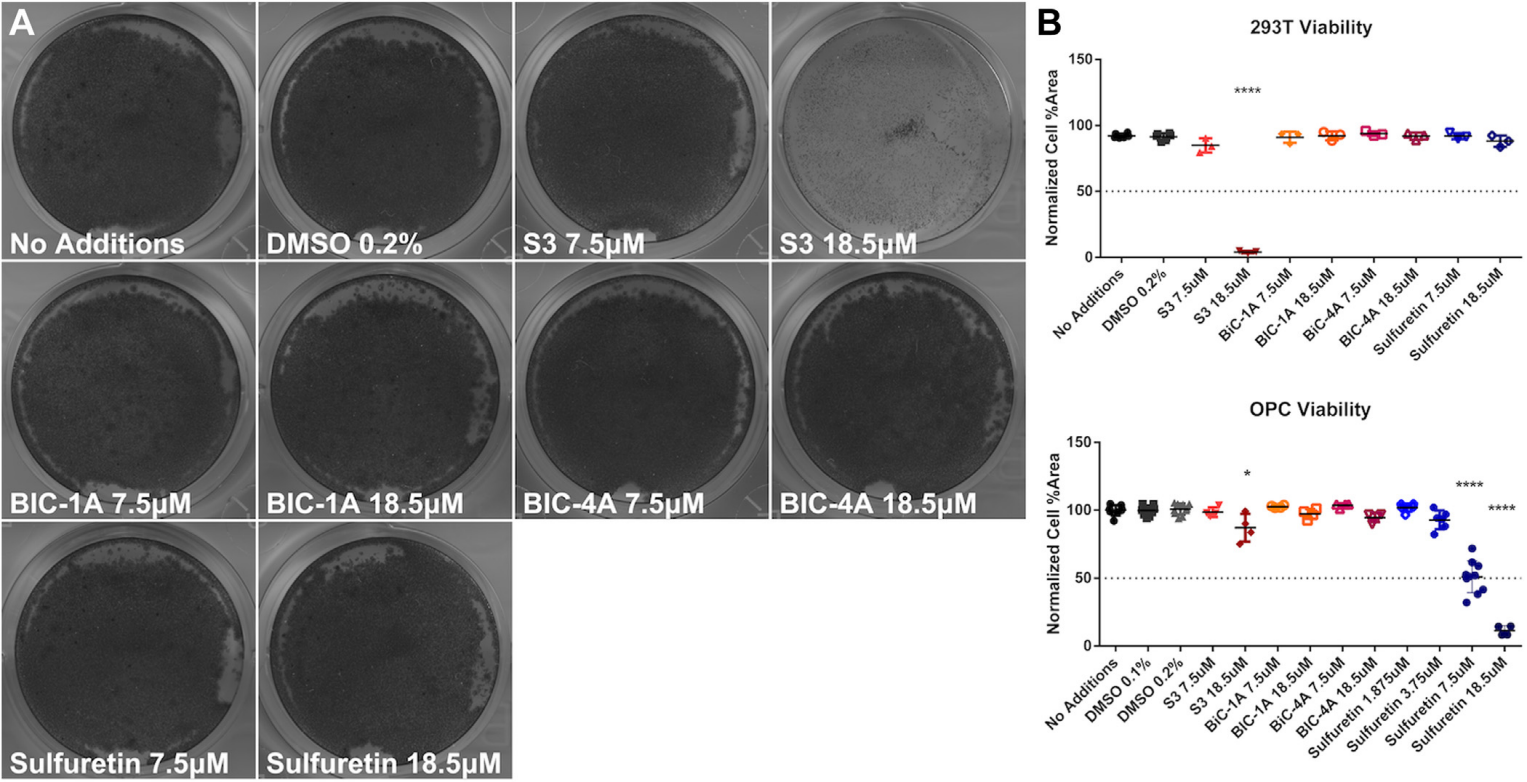
**Crystal violet viability assay of cells exposed to hyaluronidase inhibitors.***A*, representative images displaying 293T cells under normal growth conditions with indicated inhibitor doses. *B*, quantification of cell viability of 293T cells (*upper*) and OPCs (*lower*). ∗*p* < 0.05, and ∗∗∗∗*p* < 0.0001.

We next tested how our three selected inhibitors influenced cell survival and proliferation compared to S3. We grew 293T cells and OPCs in the presence of each agent and assayed cell viability using a crystal violet assay. We found that S3 greatly reduced 293T cell viability at 18.5 μM (*p* < 0.0001), while OPCs were more resistant to cell death at this concentration (*p* < 0.05; Fig. 5*A*, and compare Fig. 5*B upper* and *lower panels*). In contrast, 293T cell viability was not altered in the presence of any concentration of sulfuretin that we tested, while OPCs had reduced viability at both 7.5 μM and 18.5 μM (Fig. 5*B*, *lower panel*). BIC-1A and BIC-4A did not appreciably change cell viability in either cell type at any concentration tested.

**Figure 6:**
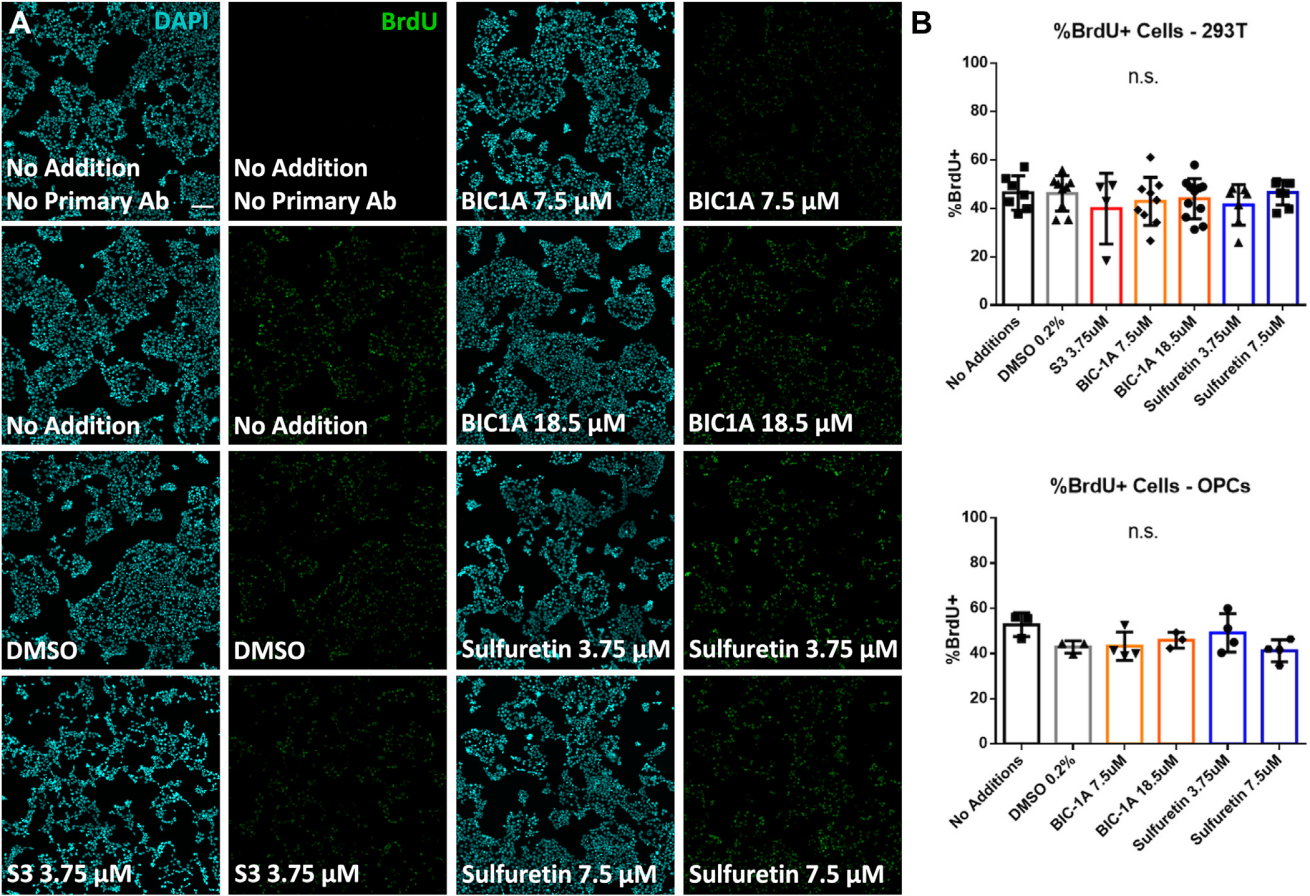
**Cell proliferation is unaffected by hyaluronidase inhibitors.***A*, representative image of 5-bromo-2-deoxyuridine (BrdU) uptake of 293T cells exposed to indicated concentrations of hyaluronidase inhibitors. *B*, percentages of BrdU^+^ 293T cells (*top panel*) and OPCs (*bottom panel*) following treatment with inhibitors. Scale bar = 100 μm. n.s. – not significant.

To determine if cell proliferation was affected by any of the CEMIP inhibitors, we measured changes in the amount of 5-bromo-2-deoxyuridine (BrdU) uptake and Ki67 expression in both cell types. We found no significant change in the percentages of Ki67^+^ (not shown, the vast majority of cells were positive in all conditions) or BrdU^+^ cells for any tested condition in both cell types (Fig. 6, *A* and *B*).

### CEMIP inhibitors promote OPC maturation

**Figure 7:**
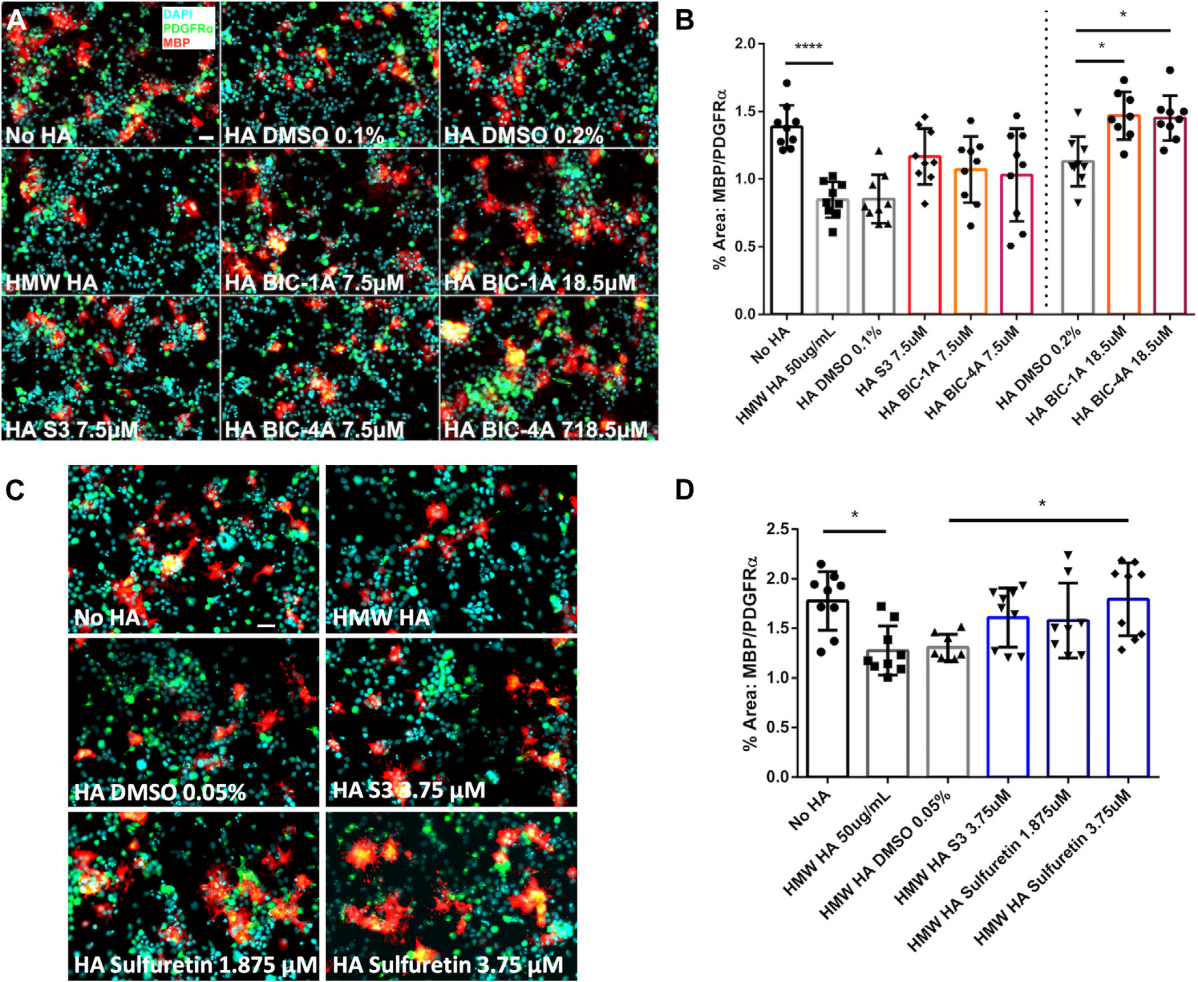
**Hyaluronidase inhibitors promote *in vitro* OPC maturation in the presence of HMW HA.***A* and *C*, representative images showing numbers of PDGFRα+ OPCs and MBP+ oligodendrocytes when exposed to HMW HA, or HMW HA with vehicle, synthetic inhibitors (*A*) or sulfuretin (*B*). *B* and *D*, quantification of OPC maturation in the presence of HMW HA, S3, and synthetically-derived hyaluronidase inhibitors. *B*, BIC-1A and BIC-4A both increase OPC maturation in the presence of HMW HA at 18.5 μM, while S3 is toxic to cells at this dose. *D*, sulfuretin is more potent than S3 at promoting OPC maturation when exposed to HMW HA. Scale bar = 30 μm. ∗*p* < 0.05, and ∗∗∗∗*p* < 0.0001.

HMW HA synthesis increases in demyelinating lesions coincident with increased hyaluronidase activity, generating HA fragments that block OPC maturation and remyelination (17, 21, 42). Inhibiting hyaluronidase activity with S3 promoted OPC maturation and functional remyelination *in vivo* (21). We therefore tested if sulfuretin, BIC-1A, or BIC-4A could also promote OPC maturation in the presence of HMW HA. We differentiated OPCs in the presence of HMW HA and each of our inhibitors. OPC maturation was determined using immunocytochemistry to differentially mark platelet-derived growth factor receptor alpha (PDGFRα) expressing OPCs and myelin basic protein (MBP) expressing mature oligodendrocytes, and the ratio of MBP to PDGFRα expression was used to determine the percentages of cells that matured (17, 21, 42). We found that at 18.5 μM, both BIC-1A and BIC-4A significantly promoted OPC maturation in the presence of HMW HA (*p* < 0.05 for both compounds; Fig. 7, *A* and *B*). Sulfuretin promoted OPC maturation at a much lower concentration (3.75 μM; *p* < 0.05, Fig. 7, *C* and *D*). In comparison, S3 was effective at between 3.75 μM and 7.5 μM and was toxic at higher concentrations (Fig. 7, *B* and *D* and data not shown).

**Figure 8:**
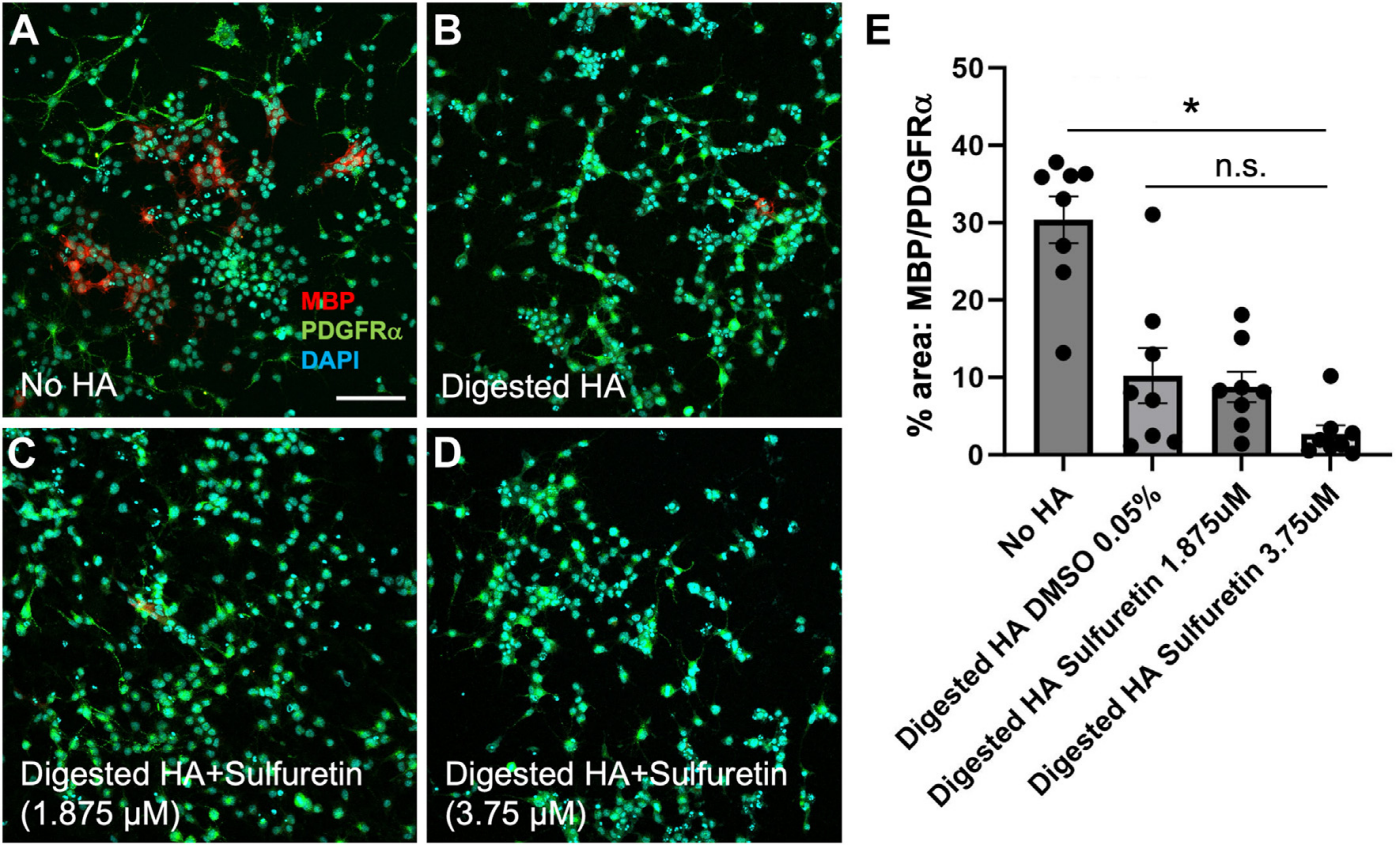
**Sulfuretin does not rescue the effects of digested HA on OPC maturation.***A*–*D*, Representative immunohistochemical analyses of MBP (*red*) and PDGFRa (*green*) expression in primary mouse OPC cultures under differentiation conditions for 72 h and treated without HA (*A*), with HMW HA digested by CEMIP (*B*), (*C*) CEMIP-digested HA+ 1.875 μM sulfurtein, and (*D*) 3.75 μM sulfurein. Scale bar = 100 μm. *E*, quantification of immunohistochemical analyses in (*A*–*D*). ∗*p* < 0.001, and n.s. = not significant.

Given that sulfuretin acts on OPC maturation at lower concentrations than the other inhibitors, we aimed to further confirm that this effect is specific to blocking the digestion of HMW HA. We therefore treated OPC cultures with CEMIP-digested HA in the presence and absence of sulfuretin, then assayed the cells for their degree of maturation into MBP-expressing oligodendrocytes. Digested HA significantly inhibited OPC maturation (Fig. 8, *A*, *B* and *E*). However, this effect was not rescued by sulfuretin at concentrations that increased OPC maturation in the presence of HMW HA (Fig. 8, *C* and *D*, R). Altogether, these data confirm that agents that can block CEMIP activity are capable of promoting OPC maturation, and support the notion that CEMIP inhibitors have the potential to be used to promote OPC maturation and remyelination in demyelinating diseases.

## Discussion

We find that multiple classes of chemical structures can act as potent inhibitors of CEMIP, a novel hyaluronidase implicated in a number of pathological processes. The inhibitors we tested differed in their abilities to block CEMIP in different cell types. The synthetic inhibitor BIC-1A had the greatest inhibitory activity in 293T cells, while sulfuretin was much more effective in primary OPCs. Although these differences may be explained, in part, by different levels of CEMIP expression and hyaluronidase activity in the different cell types, other factors, including CEMIP subcellular localization or cell type-specific differences in protein-protein interactions could explain why these agents demonstrate different activities in different cells. Importantly, these differences suggest that different classes of inhibitors may be needed for different cell types.

HA digestion products of specific sizes, and not HMW HA, have been shown to inhibit OPC differentiation (42, 43). Cells of the oligodendrocyte lineage have hyaluronidase activity (43, 44), and inhibiting this endogenous activity is a likely mechanism to restore OPC differentiation rates in the presence of HMW HA. The synthetic flavonoid S3 has previously been shown to accelerate OPC differentiation in the presence of HMW HA and to promote functional remyelination (21). Here, we have found that each of the three compounds we tested increased OPC differentiation at a greater rate than S3 at similar as well as lower concentrations, suggesting that these agents have the potential to be developed for therapeutics that target CEMIP activity in demyelinating diseases. This may be especially true for BIC-1A and BIC-4A, which are predicted to cross the blood-brain barrier (BBB) more readily than S3.

It is possible that BIC-1A, BIC-4A, and sulfuretin can influence the activities of other hyaluronidases expressed by 293T cells and OPCs. Indeed, each of these agents was originally identified using bovine testicular hyaluronidase/PH20 as a screen for extracellular hyaluronidase activity. Although we found that OPCs express multiple hyaluronidases (Fig. 4*A*), CEMIP-digested HA was sufficient to block OPC maturation (Fig. 8, *B* and *E*), and sulfuretin reversed the effects of elevated CEMIP expression on OPC maturation without affecting the expression of other hyaluronidases or HA synthases (Fig. 4*B*). In addition, CEMIP is elevated in demyelinating lesions (20). The findings with these agents, and sulfuretin in particular, therefore support the notion that their primary effect on OPC maturation was due to blocking the activity of CEMIP.

Our findings add to the list of previous flavonoids and related compounds found to act as hyaluronidase inhibitors. Sulfuretin had not previously been reported to have hyaluronidase inhibitory activity, and it blocks CEMIP activity at a lower concentration and with less toxicity than the S3 synthetic flavonoid. The structure of S3 is based on the structure of another flavonoid, apigenin, which has weak hyaluronidase inhibitory activity against a broad range of hyaluronidases (23, 24, 25). These data indicate that flavonoid structures can be modified to increase hyaluronidase inhibitory activity and specificity and that such structures could be further developed for therapeutic applications.

Phenols and polyphenols are known to inhibit the activity of hyaluronidases (45). Proposed interactive sites between hyaluronidases and these compounds have been suggested by docking studies and structure-activity relationships (22, 46, 47). Based on the results from our experiments and these previous studies, we suggest that the following structural features should be considered when evaluating flavonoids and other small molecules for anti-hyaluronidase activity: The OH groups on carbons 6 and 4′ and a carbonyl on carbon 3 should be considered essential for blocking hyaluronidase activity. A double bond between carbons 2 and 3 are less likely to be involved in activity. Finally, the OH groups on carbons 4 and 5′ are likely to be disposable for activity.

In addition to cell type-specific differences in hyaluronidase inhibitory activity, we found that different inhibitors had distinct effects on cell survival, while none of the inhibitors tested significantly influenced cell proliferation. These different effects on cell survival raise the possibility that different classes of CEMIP inhibitors are selectively toxic to certain cell types. Further investigations will determine whether such selective toxicity could be effective in treating tumors or other hyperproliferative conditions.

CEMIP expression is correlated with increased tumor metastases and poor prognosis in numerous cancers (8, 9, 10, 11, 12, 13, 48), suggesting that CEMIP hyaluronidase activity may be contributing to the ability of tumor cells to migrate and colonize other tissues. This may be due to the generation of HA digestion products. HA fragments can induce tumor angiogenesis by stimulating endothelial cell proliferation (49, 50). In addition, HA fragments can influence tumor cell migration and the expression of matrix metalloproteases that have been implicated in tumorigenesis and metastasis (5, 51). Consistent with these activities of HA digestion products that can be generated by hyaluronidases, the knockdown of CEMIP expression in tumor cells greatly reduces their ability to metastasize to the brain but not other organs (14). Our findings in tumorigenic 293T cells indicate that CEMIP inhibition does not influence tumor cell proliferation under the cell culture conditions utilized in this study. It is possible, therefore, that CEMIP activity only contributes to cancer cell metastasis but not tumor growth, although studies of the effects of blocking CEMIP activity *in vivo* are needed to confirm these findings. Nonetheless, our findings, in conjunction with previous studies, suggest that cell-type specific CEMIP inhibitors have the potential to treat metastatic disease. Further explorations into the chemistry of flavonoids and other agents similar to those identified here will reveal the potential of this strategy.

## Experimental procedures

### Identification of novel hyaluronidase inhibitors from plants

Plants were obtained from a local garden and were quickly frozen. The petals were crushed and about 500 g (125 g dry weight) were extracted three times with methanol at room temperature on a shaker for 6 to 10 h each. The solvent was filtered and evaporated under reduced pressure to obtain about 55 g of residue. 50 ml of water was added to the residue which was extracted with ethyl acetate.

The ethyl acetate extract was dried under reduced pressure to yield about 15 g. The residue was dissolved in ethyl acetate and methanol, added to silica gel and dried. It was separated using a Reveleris silica gel flash chromatography system (Buchi) using a gradient of hexane to ethyl acetate to methanol. Fractions were collected, and visualized with silica gel thin-layer chromatography after eluting with 7:1 chloroform:methanol to yield 14 fractions and fumed with iodine to identify flavonoids. The fractions were then assayed for their ability to inhibit the activity of hyaluronidase. Active fractions were pooled and chromatographed as before with flash chromatography. The active compounds were analyzed by liquid chromatography-mass spectroscopy as previously described (52) and gave the expected mass spectra. The structures of two of the compounds (sulfuretin and luteolin) were further verified using ^1^H NMR and ^13^C NMR on a 400 MHz instrument (Bruker).

### Identification and synthesis of thiocarbamate compounds

We generated a thiocarbamate library as previously described (53, 54) and screened compounds for hyaluronidase inhibitory activity as above. To generate BIC-1A and BIC-4A, 4-chloro- and 4-methylisothiocyanate were refluxed in absolute ethanol overnight. The resulting precipitate was then filtered off and recrystallized from ethanol to form crystalline solids BIC1A and BIC4A. The obtained crystalline molecules were confirmed based on their spectra data.

### Cell culture

Human embryonic kidney 293T cells were cultured in Dulbecco’s modified eagle medium (DMEM, Gibco) supplemented with 10% fetal bovine serum (FBS, Atlas Biologicals). 293T cells were passaged using 0.25% trypsin (Gibco) for cell detachment/dissociation. Primary mouse oligodendrocyte progenitor cells (OPCs) derived from oligospheres were cultured as described previously (43). Briefly, OPCs were cultured in DMEM:F12 (Gibco) supplemented with 0.1% bovine serum albumin (BSA, Fisher), recombinant platelet-derived growth factor AA (PDGF-AA, Promega), and fibroblast growth factor-2 (FGF-2), B27 supplement without vitamin A (Gibco), N1 supplement (Sigma), and Biotin (Sigma). Cells were passaged using Accutase (Invitrogen).

### CEMIP transfections and HA fragment generation and purification

CEMIP expression vector (Catalog MR217955) and empty vector (Catalog PS100001) were purchased from Origene. Transfections were performed using Fugene HD (Promega). To generate CEMIP-digested HA fragments, approximately 6 × 10^6^ 293T cells were cultured on 10 cm^2^ polystyrene dishes (Fisher) overnight, and transfected with 10 μg of plasmid the following day (1:3 DNA:Fugene HD ratio). Twenty-four hours later, culture medium was replaced with 10 mg of HMW HA (Lifecore Biomedical) in 10 ml of dye-free, serum-free, DMEM (Fisher). After 72 h, conditioned medium was harvested and HA extracted using a phenol:chloroform solution (1:1, both chemicals from Sigma). An equal volume of phenol:chloroform was added to the conditioned medium, and the solution was centrifuged at 12,000 rpm for 45 min. The aqueous layer was harvested and dialyzed in pure water using a 20,000 Da molecular weight cutoff dialysis cassette (Fisher) for 24 h, replacing the water with fresh pure water halfway through. The conditioned medium was then collected, dried overnight in a SpeedVac, and resuspended in pure water to reach a desired concentration for use in subsequent experiments. The HA yield was >95%.

### Viability assay

Cell viability was assayed using crystal violet adapted from (55). Briefly, 12-well plates (Corning) were seeded with 2 × 10^5^ 293T cells or OPCs per well. The next day, DMSO (vehicle, Fisher) or inhibitors were added to the culture medium. Cells were grown for three days, fixed in Zamboni’s fixative (4% PFA with picric acid, see (56)), and stained with 0.5% crystal violet (Fisher). Stained cells were washed in distilled water, and imaged on a scanner. Changes in viability were determined by measuring differences in percent area cell coverage between wells in ImageJ.

### Proliferation assay

5-Bromo-2-deoxyuridine (BrdU) uptake and Ki67 expression were measured to determine cell proliferation (57). Cells were seeded on coverslips (Carolina) in 24-well plates (Corning), at 1 × 10^5^ per well for both 293T cells and primary OPCs. 293T cells were plated on uncoated coverslips, while OPCs were plated on poly-L-ornithine (Sigma) coated coverslips (58). The following day, the appropriate amount of DMSO or inhibitors were added to wells. 24 h later, BrdU (Sigma) was added to wells at a final concentration of 10 μM. 293T cells were allowed to incubate for 4 h, while OPCs were incubated with BrdU for 12 h. Cells were fixed in Zamboni’s fixative (see above in Viability assay) and assayed for BrdU and Ki67 expression using immunocytochemistry (described below). Changes in the numbers of Ki67 and BrdU expressing cells were measured using ImageJ.

### HA digestion assay

CEMIP expression vector (Catalog MR217955) and empty vector (Catalog PS100001) were purchased from Origene. All transfections were completed using Fugene HD (Promega). To test for hyaluronidase activity in live cells, 6 × 10^6^ 293T cells or 3 × 10^6^ OPCs were cultured on 10 cm^2^ polystyrine dishes (Fisher) overnight for transient transfection. The following day, cells were transfected with 10 μg plasmid using Fugene HD at a ratio of 1:3 DNA to transfection reagent for 293T cells, and 1:6 for OPCs. The next day, the cell culture medium was replaced. 48 h after transfection, cells were re-plated into 24-well plates, at a concentration of 1 × 10^5^ per well for 293T cells and 2 × 10^5^ per well for OPCs.

The following day, cell culture media was replaced with dye-free, serum-free medium containing high molecular weight hyaluronic acid (Lifecore Biomedical) at 50 μg/ml with and either no other additions, dimethyl sulfoxide (DMSO, vehicle), or each inhibitor at the indicated concentrations. The culture medium was collected 72 h later and used directly in agarose gel electrophoresis assays.

HA was separated by size using agarose gel electrophoresis and analyzed by densitometry as previously described (21). Briefly, size separation was achieved in 0.5% HGT agarose gel (SeaKem) in tris-borate EDTA. The gel was stained in 0.005% Stains-all (Fisher) in 50% ethanol overnight, and washed in 10% ethanol, all in the dark. Gels were de-stained in light and then photographed. Densitometry was used to determine the percentage of CEMIP inhibition in ImageJ as described previously (21). Briefly, the pixel density of HMW HA was determined for each lane, and absorbance normalized to a 0 to 100% scale, HMW HA pixel density in the CEMIP lanes (no additions) being 0% and pixel density in the vector-transfected lanes being 100%. A CEMIP inhibition value of above 100% means the absorbance is above that seen in the vector-transfected lane.

### OPC differentiation assay

OPCs were differentiated *in vitro* as described previously (16, 20, 41, 47). OPCs were plated on poly-ornithine (Sigma) coated coverslips in 24-well plates at 3 × 10^4^ cells per coverslip. The following day, OPC culture medium was replaced with DMEM:F12 supplemented with N-acetyl cysteine (NAC, Sigma) and triiodothyronine (T3, Sigma) with and without HMW HA, vehicle, or each inhibitor at indicated concentrations. The differentiation medium was replaced daily, and cells were fixed in Zamboni’s fixative (4% PFA in phosphate buffer and aqueous picric acid, see above). Immunocytochemistry was used to determine relative amounts of PGFRα^+^ OPCs and MBP^+^ oligodendrocytes as described previously, and the percentage of area coverage of each marker was calculated using ImageJ to determine the ratio of MBP/PDGFRα expression.

### Immunocytochemistry

Immunocytochemistry was performed on fixed cells as described previously (21, 42, 43). Cells were permeabilized with 0.5% Triton X-100 (Sigma) in 1× phosphate buffered saline (PBS) for 15 min and then blocked in 5% normal goat serum (Fisher) in 1× PBS for two hours. Blocking buffer was replaced with blocking buffer and appropriate primary antibodies, and incubated at 4 °C overnight. The following day, cells were washed in 0.05% Triton X-100 in 1× PBS times for five minutes each, and then a secondary antibody was added for two hours at room temperature. Cells were then washed three times with 0.05% Triton in 1× PBS, counterstained with DAPI (Molecular Probes, 1:5000 concentration in 1× PBS for 3 min), and mounted onto glass slides (Fisher) using Fluoromount-G (Southern Biotech).

The following primary antibodies were used: PDGFRα (BD Pharmigen Catalog No. 558774, 1:100 dilution); MBP (Biolegend Catalog No. 808402, 1:800 dilution); Ki67 (Invitrogen Catalog No. MA5-14520, 1:250 dilution); BrdU (Invitrogen Catalog No. B35128, 1:250 dilution). The following secondary antibodies were used, all purchased from Invitrogen and used at a 1:1000 dilution: Goat anti-mouse AF488 (Catalog No. A11029); goat anti-mouse AF546 (Catalog No. A11003); goat anti-rabbit AF546 (Catalog No. A11035); goat anti-rat AF488 (Catalog No. A11006). Control coverslips were incubated in the absence of primary antibodies as a control for signal specificity. All antibodies had previously been validated with either peptide blocking or tests on antigen-negative cells and tissues.

### Western blotting

Cells were lysed in lysis buffer (10 mM Tris-HCl pH 8, NaCl 150 mM, EDTA 1 mM and 1% Triton X-100) supplemented with the Protease Inhibitor Mix (Sigma). Proteins were separated using SDS-PAGE (7% poly-acrylamide gel) and transferred to a nitrocellulose membrane (Thermo Scientific). Protein expression was then analyzed as previously described (42). Antibodies utilized were: rabbit anti-KIAA1199 (Sigma-Aldrich; 1:1500); mouse anti-beta-actin (Thermo Scientific; 1:10,000) as a loading control; goat anti-rabbit -HRP (Jackson ImmunoResearch; 1:10,000); and goat-anti-mouse-HRP (Jackson ImmunoResearch; 1:10,000). Blots were probed without a primary antibody to verify signal specificity.

### qPCR analysis

RNA was extracted from mouse OPC cultures with TRIzol (Invitrogen) and analyzed by qPCR as previously described (42). cDNA was prepared using a High-Capacity cDNA Reverse Transcription Kit (Applied Biosciences). qPCR analysis was performed with Power Up SYBER Green Master Mix (Applied Biosciences) using the following primers: mCyclA: forward: 5′-GGCAAATGCTGGACCAAACACAA; reverse: 5′-GGTAAAATGCCCGCAAGTCAAAAG-3′; mHyal1: forward: 5′-GCACCCTCCAACTGGGGCAG-3′; reverse: 5′-CGGTGTCGAACGTACATCTG-3′; mHyal2: forward: 5′-TCAGCTGGCGTCATCTTCTG-3′; reverse: 5′-GCCCAGGACACGTTGACTAT-3′; mCEMIP: forward: 5′-CAATGACCAAACTGGGCAGC-3′; reverse: 5′-CAGTTTGAAAACCCGGGCAG-3′; mTmem2: forward: 5′-TTGGAAACTACGTCCCTGTG-3′; reverse: 5′-CATGCAGTCTGTGGTAGGCA-3′; mHAS1: forward: 5′-GCGAGCACTCACGATCATCTT-3′; reverse: 5′-CCAGGAGTCCATAGCGATCTG-3′; mHAS2: forward: 5′-AAAGGGACCTGGTGAGACAGAA-3′; reverse: 5′-CCCATTTTTGCATGATGCAA-3′; mHAS3: forward: 5′-GCGCATTGCCTTTCCAAA-3′; reverse: 5′-TGCCACCCAGCACCTCAT-3′.

### Imaging

Stained cells were imaged using an Olympus VS 120 SlideScanner with the following objectives: 2×/0.08NA Olympus Plan ApoN, 10×/0.40NA Olympus UPlanSApo, 20×/0.75NA Olympus UPlanSApo, and 40×/0.95NA UPlanSApo. The 2× objective was used for overview scans. Cells were imaged at 20× on one plane. Analyses were performed using Fiji/ImageJ (National Institute of Health).

### Statistics

A statistically significant difference between conditions was determined using a one-way ANOVA with Tukey’s multiple comparison test for all assays performed. Statistical calculations were made in GraphPad Prism. A *p*-value below 0.05 was considered significant.

## Data availability

All data will be shared upon requests to the corresponding author.

## Conflict of interest

The authors declare that they have no conflicts of interest with the contents of this article.
